# Identification and Functional Mechanism Verification of Novel MicroRNAs Associated with the Fibrosis Progression in Chronic Kidney Disease

**DOI:** 10.1007/s10528-024-10688-7

**Published:** 2024-02-05

**Authors:** Kaiying He, Xiaochun Zhou, Jing Zhao, Hongxuan Du, Juan Guo, Rongrong Deng, Jianqin Wang

**Affiliations:** 1 Lanzhou University, Lanzhou, Gansu China; 2 Department of Nephrology, The Second Hospital & Clinical Medical School, Lanzhou University, No. 82 Cuiyingmen, Lanzhou, Gansu China; 3 Xi’an Huyi District Hospital Of Traditional Chinese Medicine, Xi’an, Shaanxi China

**Keywords:** Novel miRNAs, Chronic kidney disease, Renal fibrosis, DN, FSGS

## Abstract

Chronic kidney disease (CKD) is a serious threat to human health worldwide, and its incidence is increasing annually. A growing amount of information is emerging about the role of micoRNAs (miRNAs) in the regulation of renal fibrosis, which has aroused interest in the development of drugs that block pathogenic miRNAs or restore protective miRNAs levels. To clarify the role of miRNAs in CKD, we selected patients with significant renal fibrotic disease (diabetic nephropathy (DN) and focal segmental glomerulosclerosis (FSGS)) as the disease group, and patients with little or no renal fibrotic disease (minimal change disease (MCD) and renal carcinoma adjacent to normal kidney) as controls. Significantly differentially expressed miRNAs were obtained by human kidney tissue sequencing, subsequently verified in mice models of DN and FSGS, and subsequently inhibited or overexpressed in human renal tubular epithelial cells (HK-2) stimulated by high glucose (HG) and TGF-β1 in vitro. Therefore, the mechanism of its action in renal fibrosis was further elaborated. Finally, the downstream target genes of the corresponding miRNAs were verified by bioinformatics analysis, qRT-PCR, western blot and double luciferase report analysis. Two novel miRNAs, hsa-miR-1470-3p (miR-1470) and hsa-miR-4483-3p (miR-4483), were detected by renal tissue sequencing in the disease group with significant renal fibrosis (DN and FSGS) and the control group with little or no renal fibrosis (MCD and normal renal tissue adjacent to renal carcinoma). Subsequent human renal tissue qRT-PCR verified that the expression of miR-1470 was significantly increased, while the expression of miR-4483 was markedly decreased in the disease group (*p* < 0.05). Moreover, in vivo DN and FSGS mice models, the expression levels of miR-1470 and miR-4483 were consistent with the results of human kidney tissue. In vitro, miR-4483 was suppressed, whereas miR-1470 was induced by treatment with TGF-β1 or HG. Inhibition of miR-1470 or overexpression of miR-4483 promoted HG or TGF-β1-induced fibrosis in HK-2 cells. Further study revealed that MMP-13 and TIMP1 were the target genes ofmiR-1470 and miR-4483, respectively. Our study identifies newly dysregulated miRNA profiles related to fibrosis kidneys. miR-1470 and miR-4483 are demonstrated to participate in kidney fibrosis by regulation of MMP-13, TIMP1 respectively. Our results may represent a promising research direction for renal disorders and help identify new biomarkers and therapeutic targets for CKD.

**Supplementary Information:**

The online version contains supplementary material available at 10.1007/s10528-024-10688-7.

## Introduction

Chronic kidney disease (CKD) is a severe threat to human health worldwide, and its incidence is increasing annually (Zhang et al. 2012; Mahtal et al. 2022; Saal and Harvey 2009). In patients with CKD, renal fibrosis is the leading cause of renal function loss and is considered a major pathological feature leading to end-stage renal disease (ESRD) regardless of the initial etiology (Hallan et al. 2016; Murphy et al. 2016; Shin and Kang 2016). Renal fibrosis is characterized by increased production of a-smooth muscle actin (a-SMA) and extracellular matrix (ECM) in the interstitium (Declèves and Sharma 2014; Jha et al. 2013; Katz et al. 2010). However, the mechanisms that accelerate the progression of renal fibrosis remain largely unknown.

MicroRNAs (miRNAs) are endogenous non-coding small RNA, which largely bind to the 3′-untranslated region (3′-UTR) of the target mRNA and prevent the transcription of the mRNA from being translated into proteins. Studies have shown that miRNAs are responsible for the regulation of  > 60% of protein expression levels, thus playing significant roles in the pathophysiological processes of different diseases (Trionfini et al. 2015). As a key regulator of organogenesis, cancer and diseases, miRNAs have attracted more and more attention (Saal and Harvey 2009). In addition, some miRNAs in renal tissue play a significant role in the development of renal fibrosis (Cao et al. 2021). Further understanding of their relationship can provide a new therapeutic target for treating renal fibrosis.

A growing amount of information is emerging about the role of miRNAs in the regulation of renal fibrosis, which has aroused interest in the development of drugs that block pathogenic miRNAs or restore protective miRNAs levels. For example, increased expression of miR-21 can be detected in renal tubules and glomerulus of humans and animals with kidney disease (Denby et al. 2011). Meanwhile, the knockdown of miR-21 has a significant protective effect on renal fibrosis in mice or renal tubular epithelial cells (TECs) and mesangial cells (MCs), which significantly reduces the accumulation of ECM and α-SMA, thereby alleviating the degree of fibrosis (Liu et al. 2016; Tang et al. 2019; McClelland et al. 2015). In recent years, anti-miR-21 therapy has demonstrated its ability to ease renal injury and fibrosis in diverse animal models such as acute kidney injury (AKI) and Alport syndrome (Xu et al. 2017; Pushpakumar et al. 2021). Therefore, a potential new therapy of miR-21 for the treatment of renal fibrosis has entered phase I clinical trials (Yheskel and Patel 2017). In addition, renal fibrosis was significantly attenuated by anti-miR-214 treatment in mice prior to unilateral ureteral obstruction (UUO) modeling, so miR-214 antagonism may be a novel approach for the treatment of renal fibrosis (Denby et al. 2014).

Multiple studies have shown that a number of miRNAs are involved in the pathological process of fibrosis in CKD, so they may be valuable therapeutic targets of CKD (Mahtal et al. 2022; Cerqueira et al. 2022). However, further studies are needed to investigate the regulatory role of miRNAs in CKD, and these miRNAs could be developed as novel therapeutic medicine. Therefore, in this study, we investigated the miRNAs involved in fibrosis progression of CKD and identified their potential as therapeutic targets for fibrosis of the kidney in CKD.

## Materials and Methods

### Patient Specimens

Fresh renal biopsy samples were collected from patients with pathologically confirmed MCD, FSGS, or DN (*n* = 4 in each group) for microarray analysis. Four healthy kidney tissues from donor biopsy without obvious injuries were used as controls. Formalin-fixed paraffin-embedded (FFPE) renal biopsy specimens from patients with MCD, FSGS, DN, and healthy donor kidneys (*n* = 4 in each group) were used for further validation and location study. Patient’s inclusive and exclusive criteria are listed in Supplementary Table 1. The clinical data including age, gender, serum creatinine, estimated glomerular filtration rate (eGFR), hospital number, pathological number, and nephropuncture time were obtained at the time of renal biopsy. These data are presented in Table 1 and Supplementary Table 2. All patients were granted written informed consent to take part in the research. Additionally, the protocol was ethically approved by the Institutional Review Board of The First Affiliated Hospital of Sun Yat-sen University.

**Table 1:** Clinical characteristics of MCD, FSGS, DN patients

Group	Number	Gender	Age	Serum creatinine (μmol/L)	eGFR (mL/min/1.73m^2^)
MCD	4	2 M, 2F	28.8 ± 5.9	67.3 ± 10.4	116.4 ± 11.1
FSGS	4	3 M, 1F	34.5 ± 11.7	164.5 ± 76.6	49.2 ± 16.8
DN	4	2 M, 2F	45.5 ± 8.7	319.8 ± 162.1	25.0 ± 21.6
Normal	4	3 M, 1F	–	–	–

*MCD*: minimal change disease, *FSGS*: focal segmental glomerulosclerosis, *DN*: diabetic nephropathy, *M* male, *F* female, *eGFR* estimated glomerular filtration rate)

### Mice Models of DN and FSGS

All the experiments relating to animals were performed after The First Affiliated Hospital of Sun Yat-sen University Institutional Ethical Committee's approval and under the strict adherence to the National Institutes of Health Guide for laboratory animals' care and use.

DN model: db/db mice and db/m mice (*n* = 30 per group, male, 6–8 weeks) were purchased from Nanjing University Bio-model Company and fed with a normal diet. Body weight and blood glucose were detected once a month from the beginning, and 24-h urine was collected in the metabolic cage at 8w, 12w, 16w, 20w and 24w to detect urinary creatinine, urinary protein, and urinary albumin/creatinine in mice. A certain number of db/db mice and db/m mice were sacrificed at 12w, 16w, and 24w, respectively (*n* = 10 per group).

FSGS model: Healthy 8-week-old balb/c mice were purchased from Beijing Weitong Lihua Animal Center and randomly divided into the FSGS model and sham group (*n* = 8 per group). The mice in the model group were injected with Adriamycin (12.5 mg/kg, Sigma) twice into the tail vein of the mouse. At the same time, mice in the control group were injected with the same amount of normal saline through the tail vein. 24-h urine was collected every two weeks after injection. Subsequently, the urinary creatinine, urinary protein, and urinary albumin/creatinine were determined. Mice were sacrificed four weeks after the last doxorubicin treatment, and their kidneys were harvested for further analysis.

All the animals were anesthetized by intraperitoneal injection of tribromoethanol at the corresponding time points and sacrificed by taking blood from the heart.

### MTT Assay

Cell viability was determined by the 3-(4,5-dimethylthiazol-2-yl)-2,5-diphenyltetrazolium bromide(MTT; Sigma, St Louis, USA) colorimetric assay. HK-2 cells were cultured in 96-well plates, then subjected to various stimulations and incubated for different hours. Subsequently, cells were washed with phosphate-buffered saline (PBS) and incubated in MTT solution for three hours. The absorbance was measured at 490 nm to determine the cell viability by a microplate reader (BioTek, USA).

### RNA Isolation and Microarray Profiling

Total RNA was extracted from renal biopsies using Tri-Reagent (Invitrogen, Carlsbad, CA) and miRNeasy Mini Kit (Qiagen, Germany) according to the manufacturer's instructions. 1 μg total RNA of each sample was characterized using the miRCURY^TM^ Hy3^TM^/Hy5^TM^ Power Labeling Kit, and hybridization was performed on a miRCURY^TM^LNA array with 3100 capturing probes. These samples were scanned by the Axon GenePix 4000B microarray scanner and then imported into GenePix Pro 6.0 software to align the grid and extract data. The mean value of replicated miRNAs was taken, and the miRNAs with intensity ≥ 30 in all samples were selected for normalization factor calculation. The data expressed were normalized using the Median normalization. After normalization, major differentially expressed (DE) miRNAs were detected by Volcano Plot filtering. Finally, different miRNA expression profiles of samples were displayed by hierarchical clustering. For expression analysis, miRNAs were classified as differentially expressed if the fold change is > 1.5 and the *p*-value is < 0.05.

### In SituHybridization

In order to detect the expression levels and localization of miR-1470 and miR-4483 in the kidney, in situ hybridization was performed in control, MCD, FSGS and DN renal FFPE sections. Specific LNA-digoxigenin labeled miR-1470 probe (5′-GCCCTCCGCCCGTGCACCCCG-3'), miR-4483 probe (5′-GGGGTGGTCTGTTGTTG-3'), and Negative Control probe (5′-GaactGGGGTGCGTGTGTGAT-3′) were used (Roche Diagnostics, IN).

### Cell Culture and miRNAs Transfection

HK-2 cells were cultured in Dulbecco's modified Eagle's medium/F12 medium(Life Technologies, Carlsbad, CA) with 10% fetal bovine serum (FBS), 100-ug/mL penicillin and streptomycin (Life Technologies) at a 37 °C incubator with 5% CO2. In the six-well plate, in line with the instructions of ribo FECTTMCP transfection reagent,50 nM miR-1470, miR-4483 mimics and mimic negative control, 100 nM miR-1470, miR-4483 inhibitor and inhibitor negative control were transfected into HK-2 cells respectively, and the transfected cells were obtained at the designated time point to detect microRNA levels and expression of fibrosis factors. (The sequence of microRNA mimic, inhibitor, mimic NC and inhibitor NC are shown in Supplementary Table 3.) For the TGF-β1 or high glucose (HG) treated experiment, cells were cultured in the presence or absence of 10 ng/mL recombinant human TGF-β1 (R&D Systems, MN) or 30 mM HG for various times.

### Quantitative Real‑Time Polymerase Chain Reaction (qRT-PCR)

Total RNA was isolated from FFPE sections of MCD, FSGS, DN, and normal kidney biopsies using the RecoverAll™ Total Nucleic Acid Isolation Kit for FFPE (Invitrogen, USA) following the instructions. Meanwhile, the total RNA was extracted from fresh mouse kidney cortex and HK-2 cells using the TRIZOL Reagent Kit (Invitrogen, USA). For miRNAs and mRNAs, total RNA (1 μg) was reverse transcribed into cDNA using Roche Transcriptor First Strand cDNA Synthesis Kit according to the manufacturer's protocol. QRT-PCR was performed on the ABI 7900 system using the Roche FastStart Universal SYBR Green Master (ROX). All data were normalized to U6 or β-actin expression. The relative RNA expression was calculated with the method of 2^−ΔΔCt^. These primers are listed in Supplementary Table 4.

### Western Blotting

Proteins isolated from mice kidney tissue samples and cultured HK-2 cells were separated by SDS-PAGE and transferred to PVDF membranes (Millipore, USA), blocked with 5% non-fat dried milk, and incubated with primary antibody at 4 °C  overnight, anti-COL-1 antibody (1:1000, SouthernBiotech, USA), anti-COL-3 antibody (1:1000, SouthernBiotech, USA), anti-a-SMA antibody (1:1000, Sigma, USA), anti-fibronectin antibody (1:1000, BD, USA), anti-Vimentin antibody (1:1000, Abcam, USA), anti-E-cadherin antibody (1:1000, BD, USA), anti-MMP-13 antibody (1:1000, Affinity, China), anti-TIMP1 antibody (1:1000, Affinity, China). After that, the secondary antibodies were used and the signals were detected by enhanced chemiluminescence (Merck Millipore, USA). The density of the bands was quantified and normalized to β-actin using Image-J software.

### Immunofluorescence Assay

The FFPE of mice kidneys or HK-2 cells were prepared by a routine procedure and incubated with anti-E-cadherin (E-CAD) antibody (1:20, BD, USA), anti-Vimentin antibody (1:20, Abcam, USA), anti-COL-1 antibody (1:20, SouthernBiotech, USA), anti-COL-3 antibody (1:20, SouthernBiotech, USA) and anti-COL-4 antibody (1:20, SouthernBiotech, USA) overnight at 4 °C. The slides were then incubated with Cy3- or fluorescein isothiocyanate (FITC)-conjugated secondary antibodies for 1 h at 37 °C and mounted with the anti-fade medium using DAPI to visualize the nuclei. The slides were visualized on a ZEISS confocal microscope for each group (*n* = 3).

### Histology Staining

The FFPE of renal sections from mice were prepared by a routine procedure. The glomerular collagen deposition, interstitium, glomerular volume, mesangial cells, and mesangial matrix were detected by H&E, Masson, PAS, and PASM staining in 4 μm paraffin sections. After histologic staining, neutral balsam was used for sealing, and representative images were seized using Leica Microscopy for each group (*n* = 3).

### Luciferase Reporter Assays

The 3′-UTRs for MMP13 and TIMP1 were PCR-amplified from genomic DNA. PCR primers were used to amplify the MMP13 and TIMP1 (primer sequences are listed in Supplementary Table 4). The amplified 3′-UTRs were cloned to the downstream of the luciferase coding region in the pGL-3 control (Clontech). HK-2 cells were cultured in 24-well plates for 24 h before transfection. 200 ng of the reporter plasmid was co-transfected with the control Renilla-luciferase plasmid using Lipofectamine 3000 (Invitrogen). To assess the effect of miR-1470, miR-4483 on reporter activity, 20uM of synthetic miRNA mimics (FuNeng GuangZhou) were cotransfected. Then, cells were collected 48 h after transfection and luciferase activities were analyzed by Dual-Luciferase Reporter Assay (Promega, USA). Renilla-luciferase activity was normalized to Firefly luciferase expression of every sample. Data were expressed as mean ± SEM of three independent experiments.

### Statistical Analysis

The experimental results are expressed in the form of mean ± SEM. The results of at least three independent and repeated experiments were taken, and the data were analyzed using GraphPad Prism software. The independent samples t-test was used, and for comparison between multiple groups, the LSD method was used in one-way method ANOVA. *P* < 0.05 indicated that the difference is statistically significant.

## Results

### Clinical Indicators of MCD, FSGS and DN Patients

The clinical characteristics of MCD, FSGS and DN patients are summarized in Table 1. The serum creatinine and eGFR levels for the MCD, FSGS and DN groups were 67.3 ± 10.4 μmol/L, 164.5 ± 76.6 μmol/L, 319.8 ± 162.1 μmol/L and 116.4 ± 11.1, 49.2 ± 16.8, 25.0 ± 21.6, respectively, indicating progressive deterioration of renal function in MCD, FSGS and DN patients.

### miRNA Expression Profile in Kidney Samples from DN, FSGS and MCD Patients

**Fig. 1:**
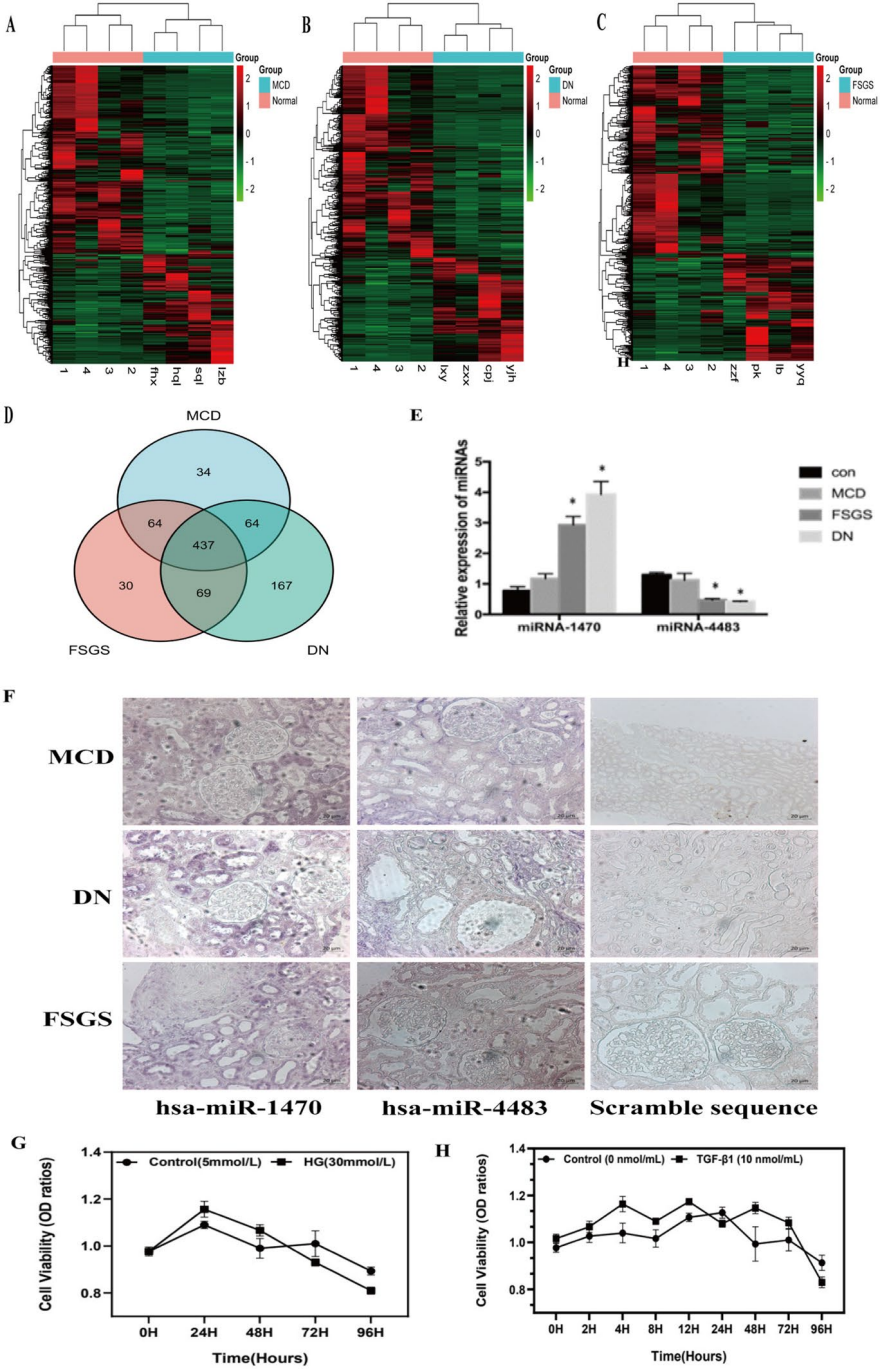
**A**–**C**: Number of DE miRNAs in kidney biopsy tissues of MCD, DN, FSGS. The expression of these miRNAs in MCD, DN, FSGS are illustrated in the heatmaps (*n* = 4 in each group). The miRNAs with fold change > 1.5 and *p*-value < 0.05 for expression in MCD, DN, FSGS patients compared to normal control were considered as differentially expressed (DE). **D**: Venn diagram has shown the number of DE miRNAs in MCD, DN, FSGS groups. **E**: ISH data using human FFPE sections show that both miR-1470 and miR-4483 were mainly expressed in tubular epithelial cells. **F**: qRT-PCR shows the expression levels of miR-1470 and miR-4483 in human FFPE sections of MCD, FSGS, DN and normal kidney tissues from healthy donor. **G**: Cell viability stimulated by HG stimulation at different concentrations (NG, HG) for 0 h, 24 h, 48 h, 72 h and 96 h. H: Cell viability stimulated by TGF-β1 stimulation at 10 ng/mL concentrations for 0 h, 2 h, 4 h, 8 h, 12 h, 24 h, 48 h, 72 h and 96 h. MCD: minimal change disease, FSGS: focal segmental glomerulosclerosis, DN: diabetic nephropathy, Con: normal kidney tissues from healthy donor. *p < 0.05 versus control group

Kidney tissues from DN, FSGS and MCD patients of suitable age and gender were selected for analysis according to the results of renal pathology from the Department of Pathology of the First Affiliated Hospital of Sun Yat-sen University (Supplementary Table 3 for inclusive and exclusive criteria). A total of 16 fresh kidney biopsies were used for miRNA expression profiling by microarray screening. The majority of miRNAs were found differentially expressed (DE) in DN, FSGS, MCD patients compared with control patients (the DE heat map is shown in Fig. 1A–C). As shown in the table, by comparison of the DE miRNA profiles among these three groups, 437 miRNAs were regarded to be commonly dysregulated as compared with the control groups (Fig. 1D). According to the above detection results, miR-1470 is the most upregulated microRNA among the DE microRNAs, whereas miR-4483 is the most downregulated microRNA. The detection results of these two miRNAs in the chips of patients with DN, FSGS, and MCD are shown in Table 2.

**Table 2:** MiR-1470 and miR-4483 in FSGS, DN and MCD compared with the normal group

	FSGS FC	*p*-value	DN FC	*p*-value	MCD FC	*p*-value
Up-regulated						
has-miR-1470-3p	63.761	0.046	117.892	0.041	–	–
Down-regulated						
hsa-miR-4483-3p	0.034	0.000	0.166	0.003	–	–

*FC* fold change

### Validation of miR-1470 and miR-4483 in DN, FSGS and MCD Patients

MiR-1470 and miR-4483 were established to change consistently among the three groups by qRT-PCR. The expression of miR-1470 was mainly upregulated in the FSGS, DN groups compared with the control and MCD groups, while miR-4483 was downregulated in the diseased kidneys (Fig. 1F). The results of in situ hybridization (ISH) using human FFPE sections showed that both miR-1470 and miR-4483 were primarily expressed in human tubular epithelial cells (Fig. 1E).

### HK-2 Cell Viability Under HG and TGF-β1 Stimulation

MTT assay was performed to detect the cell viability of HK-2 cells under HG (30 mM) and TGF-β1 (10 ng/mL) stimulation at different time points of stimulation. As can be seen from Fig. 1G, H, the viability of cells was fair from 0 h–72 h, while it significantly declined at 96 h under the stimulation of HG. Similarly, the activity of TGF-β1 stimulated cells significantly reduced after 96 h of stimulation. Therefore, the duration of the stimulus of HG and TGF-β1 was set for 72 h.

### Expression of miR-1470 and miR-4483 in the Kidney of DN, FSGS Mice Models

#### Biochemical Parameters of Blood and Urine in DN, FSGS Mice Models

**Fig. 2:**
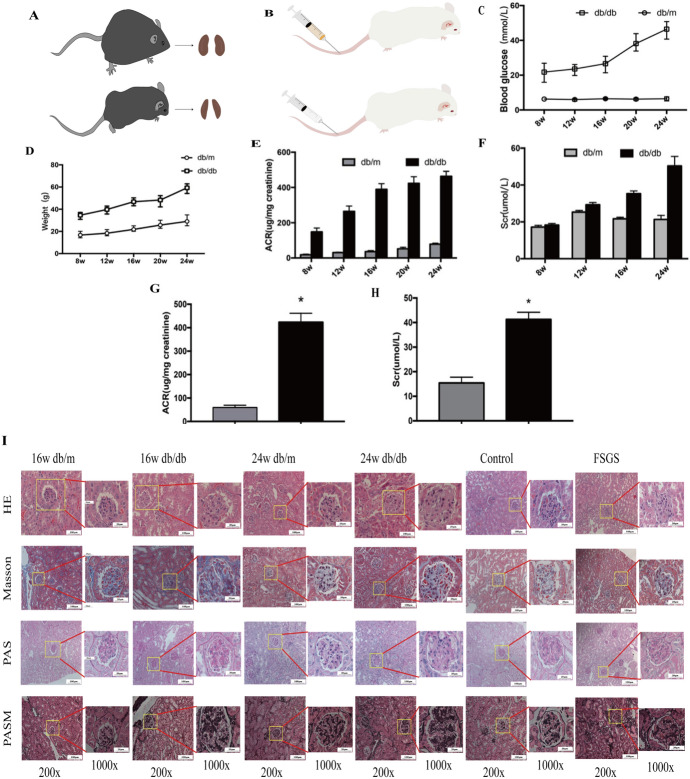
**A**: Simulated images of DN mice (16w db/db, 24w db/db) and db/m mice. **B**: Simulated images of FSGS mice and control mice. **C**: Comparison of weight between db/db mice and db/m mice at different weeks of age. **D**: Comparison of blood glucose between db/db mice and db/m mice at different weeks of age. **E**: Comparison of urinary albumin/creatinine(ACR) between db/db mice and db/m mice at different weeks of age. **F**: Comparison of serum creatinine (Scr) between db/db mice and db/m mice at different weeks of age. **G**: Comparison of urinary albumin/creatinine(ACR) between FSGS mice and control mice. **H**: Comparison of serum creatinine (Scr) between FSGS mice and control mice. **I**: H&E, Masson, PAS, PASM staining was used to observe the pathological change in kidney tissues among groups (magnification times: 200 × and 1000 ×)

During animal modeling, two db/db mice died for unknown reasons and were removed from the experimental group. Simulate images of DN and FSGS model mice are shown in Fig. 2A, B, it can be seen that at the corresponding week age, the body weight and kidney tissue of db/db mice are larger than that of control mice. Figure 2C–F show that the body weight, blood glucose, urinary albumin/creatinine ratio (ACR), and serum creatinine (Scr) of db/db mice increased gradually compared with that of db/m mice from 8 to 24w. Meanwhile, the Scr and ACR of the FSGS group were also increased compared with the control group, as shown in Fig. 2G, H.

#### Pathological Changes of Renal Tissue in DN, FSGS Mice Model

As can be seen from the staining of HE, Masson, PAS and PASM in Fig. 2I, db/db mice at 16w and 24w experienced glomerulus hypertrophy, severe enlargement and widening of the mesangial matrix, resulting in nodular sclerosis, K-W nodules and more obvious collagen deposition in the glomerulus and renal interstitium compared with 16w and 24w db/m mice. In addition, mesangial and mesangial matrix hyperplasia, glomerulosclerosis, mesangial matrix broadening, diffuse basement membrane thickening, and glomerular and renal interstitial collagen deposition were obvious in the FSGS model group compared with control mice.

#### The mRNA and Protein Levels of Renal Fibrosis Indices in DN and FSGS Mice

**Fig. 3:**
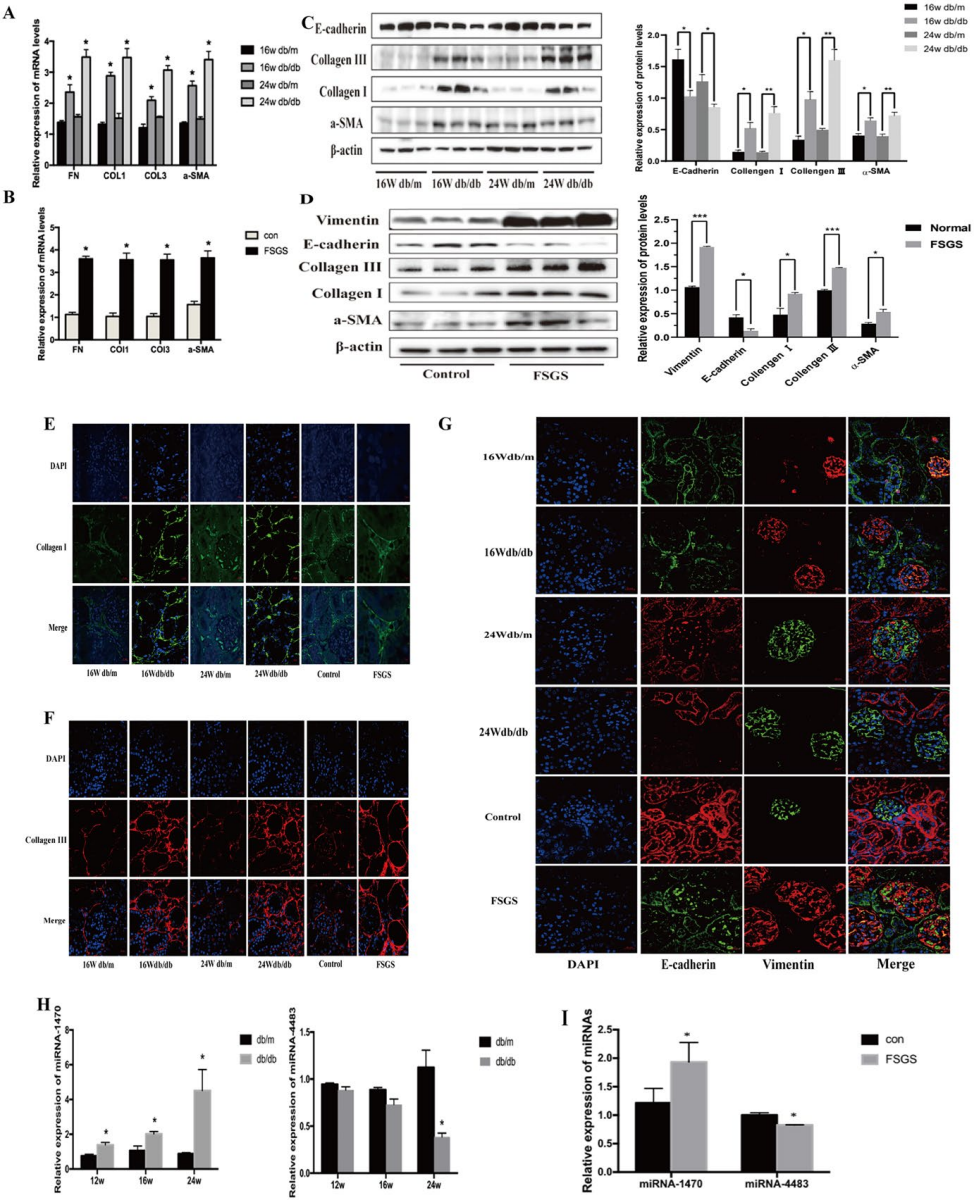
**A**: QRT-PCR was used to detect the mRNA levels of fibrosis markers (including FN, COL1, COL3 and a-SMA) among the four groups. **B**: QRT-PCR was used to detect the mRNA levels of fibrosis markers (including FN, COL1, COL3 and a-SMA) among the two groups. **C**: Western blot assay and quantitative analysis was used to detect the protein levels of fibrosis markers (including E-CAD, COL1, COL3, a-SMA, Vimentin) among the four groups. **D**: Western blot assay and quantitative analysis was used to detect the protein levels of fibrosis markers (including E-CAD, COL1, COL3, a-SMA, Vimentin) among the two groups. **E, F, G**: Immuno-fluorescence of E-CAD, COL1, COL3 and Vimentin among the six groups. **H, I**: QRT-PCR was used to detect the levels of miR-1470 and miR-4483 in the kidney of DN and FSGS, respectively. *P < 0.05, **P < 0.01, ***P < 0.001

In order to confirm the successful modeling of DN and FSGS mice, the kidney tissues of db/db, db/m, FSGS and control mice were taken to extract RNA and protein to verify the mRNA and protein expression levels of the related fibrosis indicators. As shown in Fig. 3A, B, qRT-PCR results revealed that the mRNA expression levels of Fibronectin (FN), Collagen I (COL1), Collagen III (COL3), and a-SMA in 16w, 24w db/db mice and the FSGS group were remarkably higher than those in db/m mice of the same week and the control mice. Moreover, as shown in Fig. 3C, western blot and semi-quantitative analysis were also performed to verify the protein expression levels of COL1, COL3 and Vimentin were significantly higher, while the level of E-cadherin (E-CAD) was lower in 16w and 24w db/db mice than in db/m mice of the same week. At the same time, the protein expression of Vimentin, COL1, COL3, and a-SMA in FSGS mice were elevated than those in control mice, and the level of E-CAD was decreased, as shown in Fig. 3D. Furthermore, the immunofluorescence results in Fig. 3E–G indicate that the protein expression of COL1, COL3 and Vimentin are higher, the level of E-CAD is lower in 16w, 24w db/db mice and FSGS mice than in db/m mice of the same week and the control group.

#### Validation of miR-1470 and miR-4483 Expression Levels In Vivo

In conclusion, the renal fibrosis mice models of DN and FSGS were established successfully. As shown by qRT-PCR in Fig. 3H, I, miR-1470 was up-regulated in DN and FSGS kidneys, while miR-4483 was down-regulated, which is in complete agreement with the results from human samples.

#### Role of miR-1470 and miR-4483 in HG-Induced Fibrosis in HK-2

**Fig. 4:**
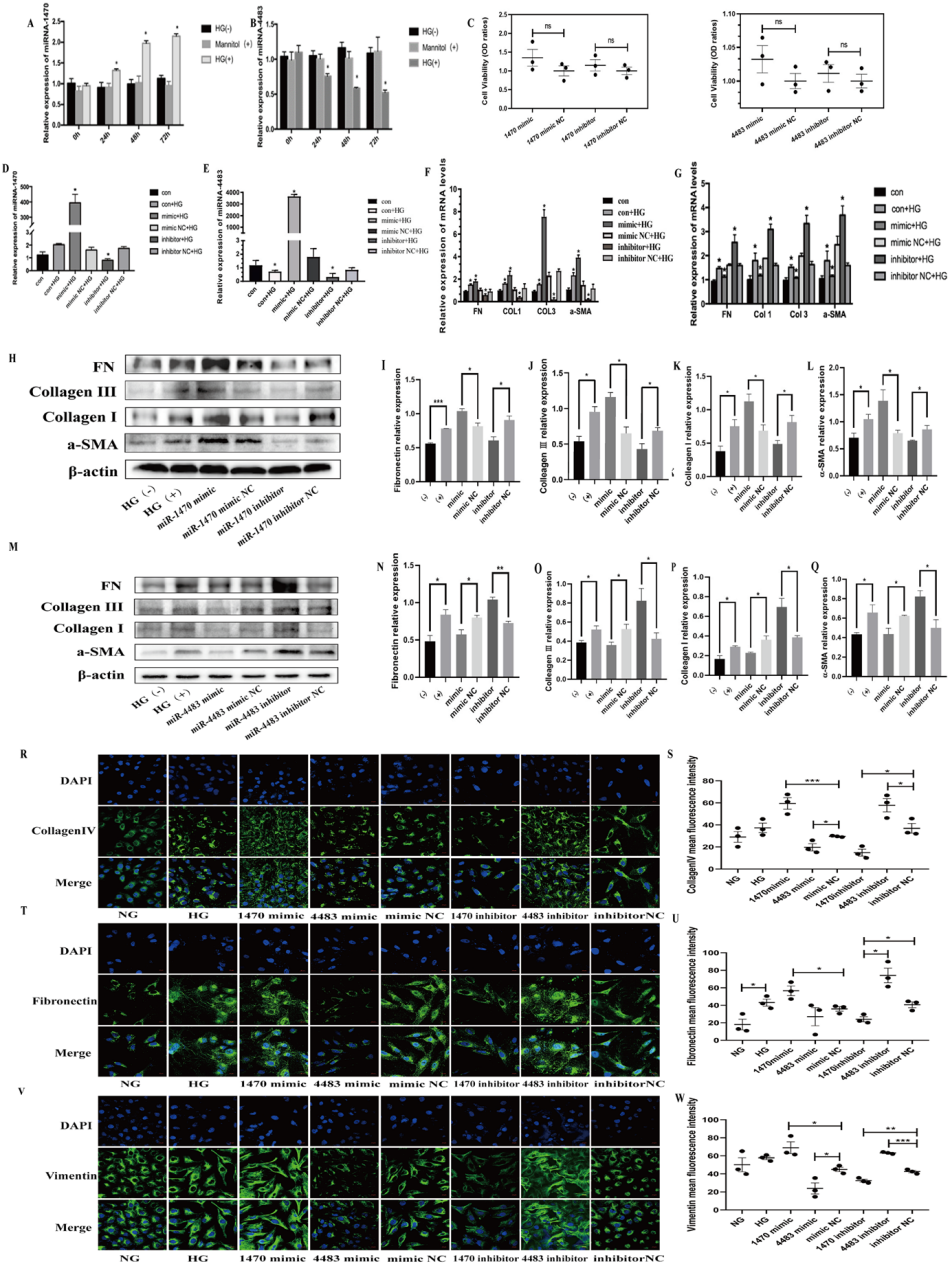
**A, B**: QRT-PCR was used to detect the levels of miR-1470 and miR-4483 in HK-2 cells stimulated by HG (30 mmol/L) for 0 h,24 h,48 h,72 h. **C:**Changes of cell viability under different transfection conditions. **D, E**: QRT-PCR was used to detect the levels of miR-1470 (**D**) and miR-4483 (**E**) in HK-2 cells after overexpression or inhibition of miR-1470 and miR-4483 under HG stimulation. **F, G**: QRT-PCR was used to determine the mRNA levels of fibrosis markers (including FN, COL1 COL3 and a-SMA) after overexpression or inhibition of miR-1470 (**F**) or miR-4483 (**G**) among the five groups. **H, I, J, K,L**: Western blot and quantitative analysis were used to detect the protein levels of FN, COL1, COL3 and a-SMA among HG (−), HG (+), miR-1470 mimic + HG, miR-1470 mimic NC+HG, miR-1470 inhibitor+HG, miR-1470 inhibitor NC+HG groups. **M, N, O, P, Q**: Western blot and quantitative analysis were used to determine the protein levels of FN, COL1, COL3 and a-SMA among HG (−), HG (+), miR-4483 mimic+HG, miR-4483 mimic NC+HG, miR-4483 inhibitor+HG, miR-4483 inhibitor NC+HG groups. **R, S, T, U, V, W**: Immunofluorescence of collagen IV, FN, Vimentin and quantitative immunofluorescence analysis of collagen IV, FN, Vimentin in HK-2 with HG, HG+miR-1470 mimic, HG+miR-1470 mimic NC, miR-1470 inhibitor, miR-1470 inhibitor NC, HG+miR-4483 mimic, HG+miR-4483 mimic NC, miR-4483 inhibitor, miR-4483 inhibitor NC transfection, respectively. ns: no significance,*P < 0.05, **P < 0.01, ***P < 0.001

In order to determine the expression of miR-1470 and miR-4483 in HG-induced fibrosis of HK-2, cells were collected from normal glucose (5.5 mmol/L, NG), NG+mannitol hyperosmotic (5.5 mmol/L+24.5 mmol/L mannitol, NM), and HG (30 mmol/L) for 0 h, 24 h, 48 h, and 72 h after synchronous treatment for qRT-PCR. As shown in Fig. 4A, B, the expression of miR-1470 was upregulated in a time-dependent manner after HG stimulation compared with HK-2 cultured in NG or NM medium. In contrast, the expression of miR-4483 showed an opposite trend. Then, miR-1470 mimic, miR-1470 inhibitor, miR-4483 mimic, miR-4483 inhibitor and miRNA mimic NC or miRNA inhibitor were transfected into HK-2 cells, respectively. MTT assay showed that the above transfection had no significant effect on cell viability(Fig. 4C). Under the stimulation of HG, the result of qRT-PCR showed that the levels of miR-1470 and miR-4483 in the miR-1470 mimic and miR-4483 mimic transfection group were significantly higher than that in the miRNA mimic NC group, and these levels were significantly reduced after the miRNA inhibition transfection compared with the miRNA inhibitor NC group (Fig. 4 D, E).

QRT-PCR, western blot and semi-quantitative analysis showed that the mRNA and protein levels of fibrosis markers (FN, COL1, COL3, a-SMA) augmented under HG stimulation, suggesting that HG could promote fibrosis in HK-2 (Fig. 4F–Q). The results showed that when miR-1470 was overexpressed, or miR-4483 was inhibited, the expressions of FN, COL1, COL3, a-SMA mRNA and protein levels were greatly enhanced under HG stimulation. On the contrary, when miR-1470 was inhibited, or miR-4483 was overexpressed, the expression level of these proteins declined (Fig. 4F–Q). Meanwhile, immunofluorescence results showed that under the stimulation of HG, the levels of FN, COL4 and Vimentin were increased after miR-1470 was overexpressed or miR-4483 was inhibited, whereas the expression of these proteins decreased when miR-1470 was inhibited or miR-4483 was overexpressed (Fig. 4R–W).

#### Role of miR-1470 and miR-4483 in TGF-β1-Induced Fibrosis in HK-2

**Fig. 5:**
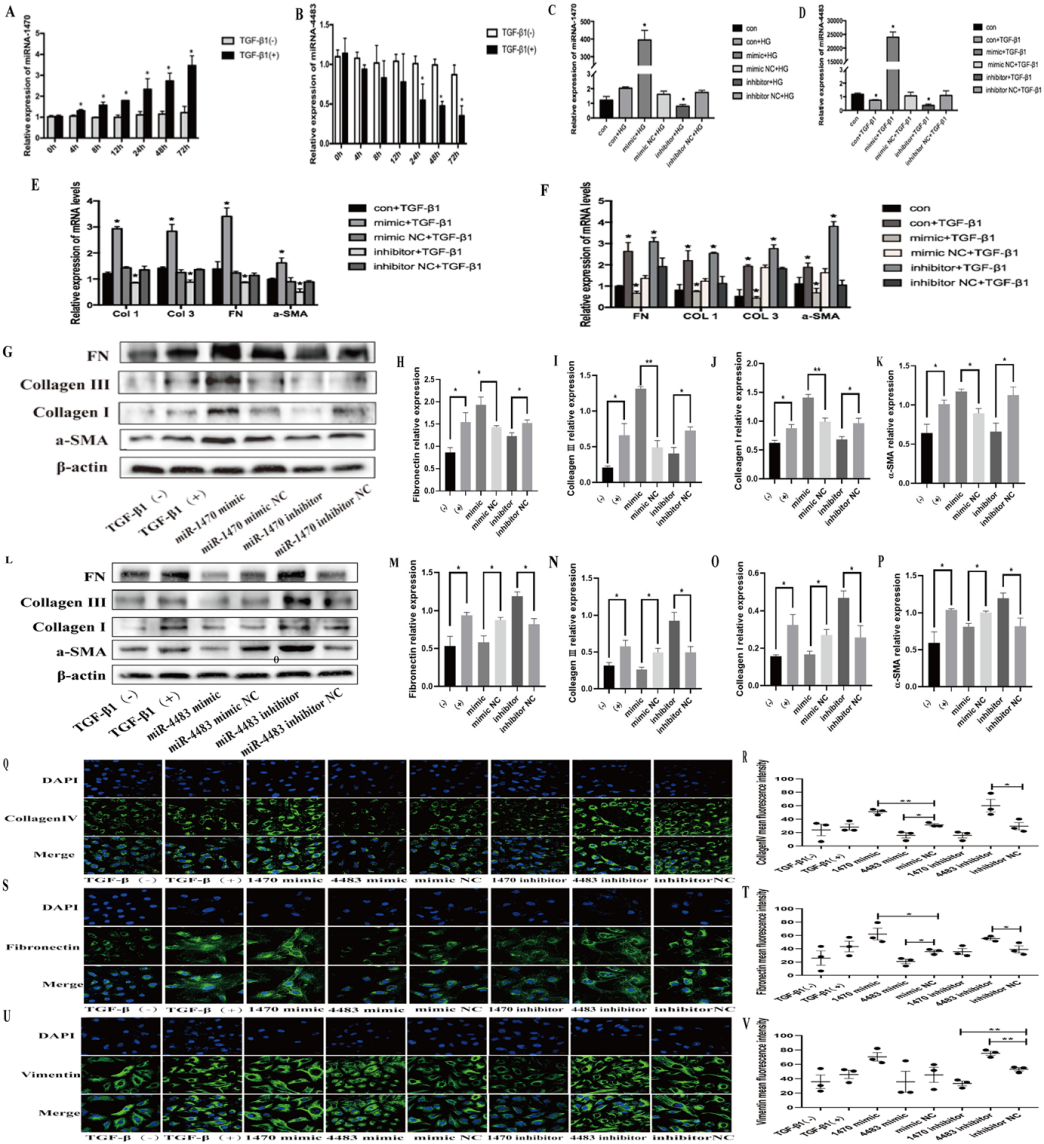
**A, B**: QRT-PCR assay was used to detect the levels of miR-1470 and miR-4483 in HK-2 cells stimulated by TGF-β1(10 ng/mL) for 0 h,2 h,4 h,8 h,12 h,24 h,48 h,72 h. **C, D**: QRT-PCR assay was used to detect the levels of miR-1470 (**C**) and miR-4483 (**D**) in HK-2 cells after overexpression or inhibition of miR-1470 and miR-4483 under TGF-β1 stimulation. **E, F**: QRT-PCR was used to detect the mRNA levels of fibrosis markers (including FN, COL1, COL3 and a-SMA) after overexpression and inhibition of miR-1470 (**E**) or miR-4483 (**F**) among the five groups. **G, H, I, J, K**: Western blot and the quantitative analysis were used to detect the protein levels of FN, COL1, COL3 and a-SMA among TGF-β1(−),TGF-β1(+), miR-1470 mimic+TGF-β1, miR-1470 mimic NC+TGF-β1, miR-1470 inhibitor+TGF-β1, miR-1470 inhibitor NC+TGF-β1. L, M, N, O, P: Western blot and the quantitative analysis was used to detect the protein levels of FN, COL1, COL3 and a-SMA among TGF-β1(−), TGF-β1(+), miR-4483 mimic+TGF-β1, miR-4483 mimic NC+TGF-β1, miR-4483 inhibitor+TGF-β1, miR-4483 inhibitor NC+TGF-β1. **Q, R, S, T, U, V**: Immunofluorescence of collagen IV, FN, Vimentin and quantitative immunofluorescence analysis of Collagen IV, FN, Vimentin in HK-2 with TGF-β1(−), TGF-β1(+), miR-1470 mimic+TGF-β1, miR-1470 mimic NC+TGF-β1, miR-1470 inhibitor+TGF-β1, miR-4483 mimic+TGF-β1, miR-4483 mimic NC+TGF-β1, miR-4483 inhibitor+TGF-β1, miR-4483 inhibitor NC+TGF-β1. *P < 0.05, **P < 0.01, ***P < 0.001

The expression levels of miR-1470 and miR-4483 in TGF-β1-induced fibrosis of HK-2 cells showed the same trend as the HG stimulation detected by qRT-PCR, as shown in Fig. 5 A–B. Similarly, qRT-QPCR results showed that the levels of miR-1470 and miR-4483 were particularly high in the mimic transfection group compared with mimic NC group, and the expression of these two miRNAs was lower in the inhibitor transfection group than in the inhibitor NC group under TGF-β1 stimulation ( Fig. 5C–D).

TGF-β1 stimulation has the same effect on promoting fibrosis as HG stimulation. QRT-PCR, western blot, and semi-quantitative analysis showed that under TGF-β1 stimulation, the mRNA and protein levels of fibrosis-related markers (FN, COL1, COL3, a-SMA) were greatly boosted after miR-1470 overexpression or miR-4483 inhibition, and the above fibrosis markers were also inhibited after inhibition of miR-1470 expression or overexpression of miR-4483 (Fig. 5E–P). Besides, immunofluorescence showed that under TGF-β1 stimulation, the levels of FN, COL4 and Vimentin increased after overexpression of miR-1470 or inhibition of miR-4483. Conversely, the expressions of the above proteins were reduced after miR-1470 inhibition or miR-4483 overexpression (Fig. 5Q–V).

#### Analysis of the Target Genes of miR-1470 and miR-4483

**Fig.6:**
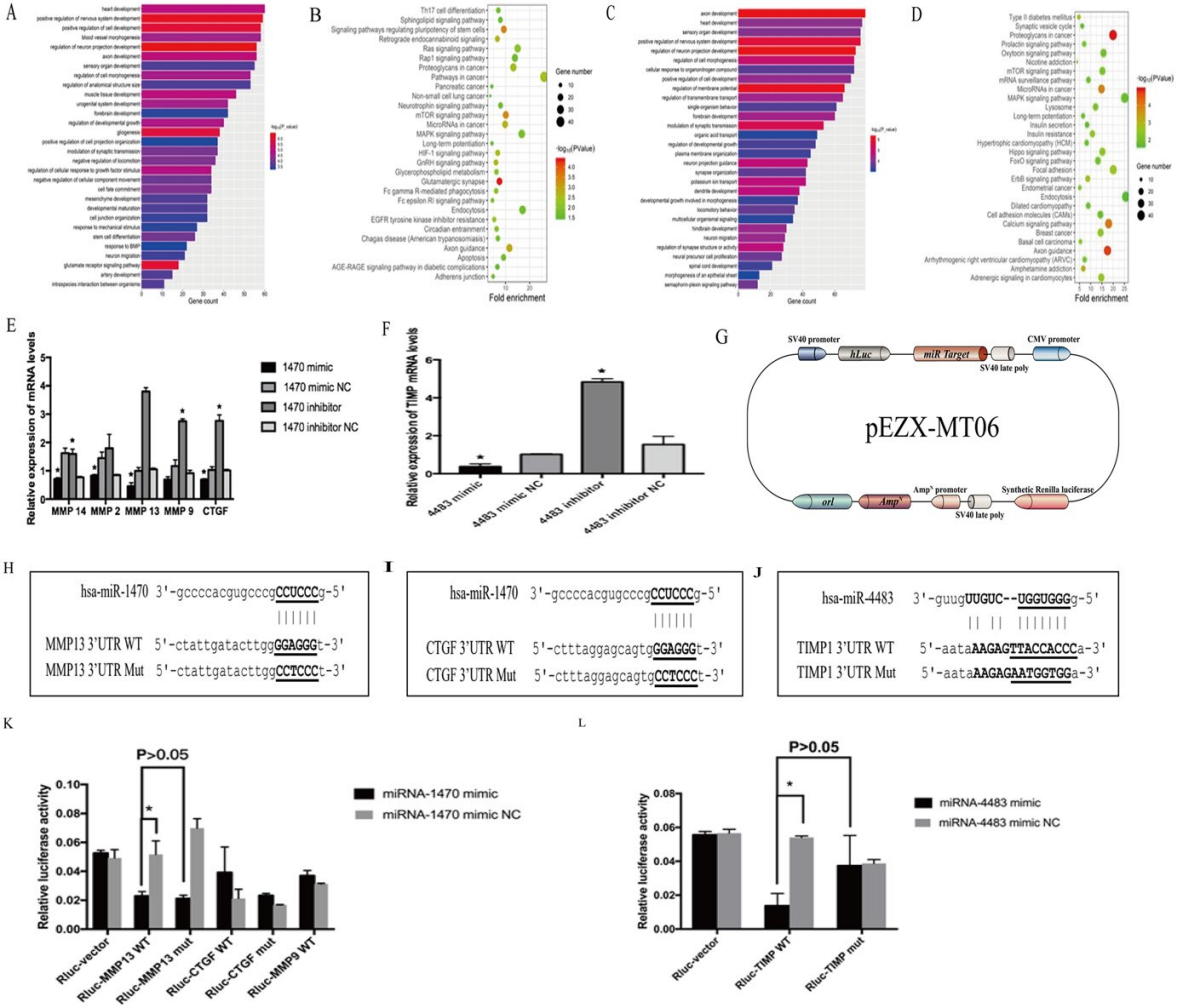
**A, B:** GO and KEGG enrichment predicted the top 30 significant target genes of miR-1470. **C, D**: GO and KEGG enrichment predicted the top 30 significant target genes of miR-4483. **E**: QRT-PCR was used to determine the mRNA expression of related downstream target genes (MMP-14, MMP-2, MMP-13, MMP-9, CTGF) under miR-1470 overexpression or inhibition conditions. **F**: QRT-PCR was used to detect the mRNA expression of the related downstream target gene (TIMP-1) under miR-4483 overexpression or inhibition conditions. **G** Simple illustration of the double luciferase gene reporter vector. **H, I, J**: The schematic luciferase reporter constructs. A mutant construct was made by replacing four nucleotides in the miRNA seed binding site of the 3'-UTR of the target gene. The mutant nucleotides were marked in bold and underlined. **K**: The luciferase reporter assay of MMP-13 3′-UTR reporter in HK-2 cells for 48 h after miR-1470 mimic transfection. **L**: The luciferase reporter assay of TIMP-1 3′-UTR reporter in HK-2 cells for 48 h after miR-4483 transfection

To characterize the potential biological functions of miR-1470 and miR-4483, the putative target genes of these two novel miRNAs were predicted by TargetScan, miRanda and then subjected to GO analysis and KEGG pathway enrichment analysis so that the downstream target genes could be predicted online (Fig. 6 A–D). Among all the genes examined in the previous step, we preliminarily screened target genes associated with fibrosis for further verification.

**Fig. 7:**
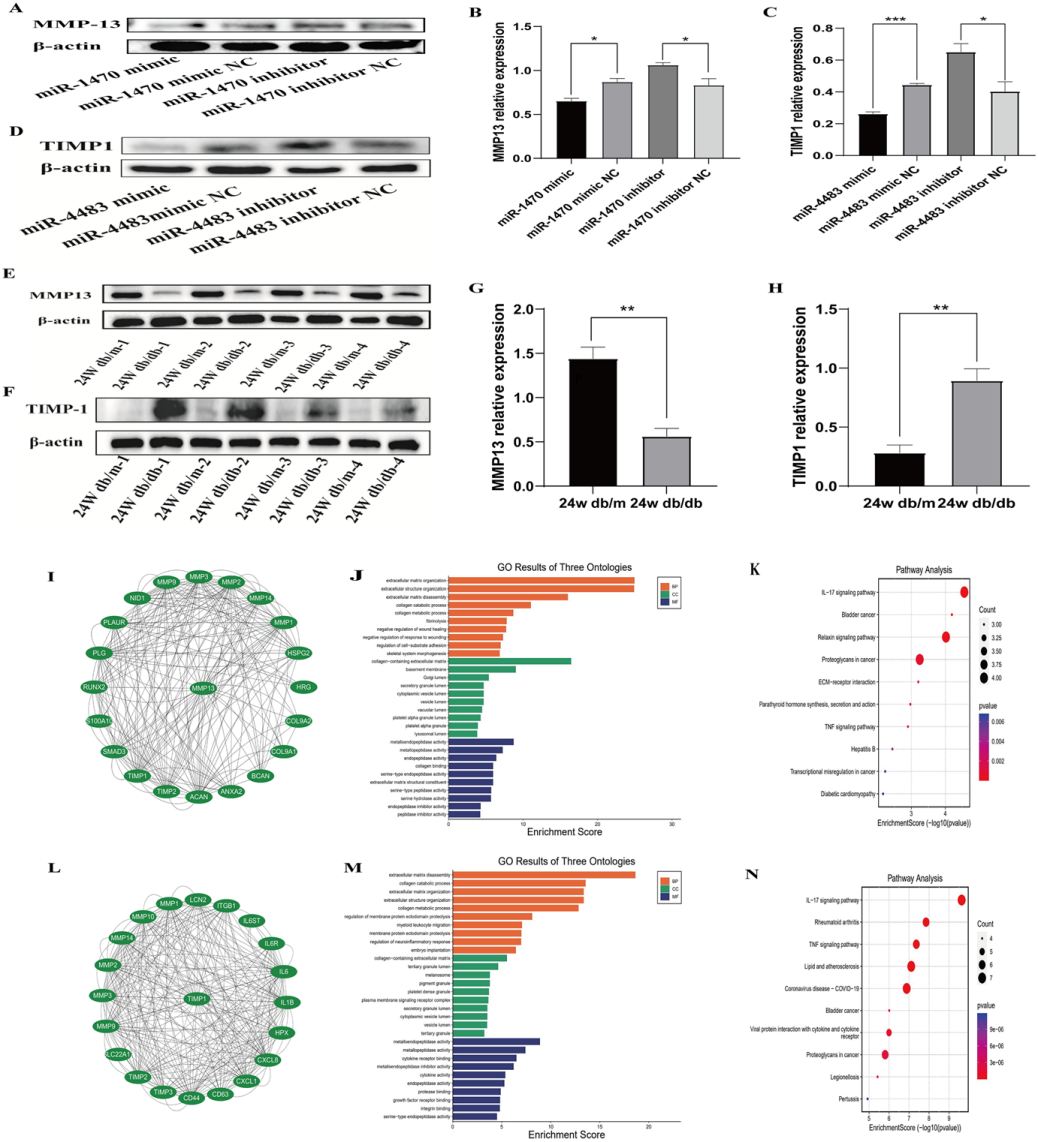
**A, B:** The protein expression and quantitative analysis of MMP-13 after transfection of miR-1470 mimic, miR-1470 mimic NC, miR-1470 inhibitor, miR-1470 inhibitor NC in HK-2 cells. **C, D**: The level of protein expression and quantitative analysis of TIMP-1 after transfection of miR-4483 mimic, miR-4483 mimic NC, miR-4483 inhibitor, miR-4483 inhibitor NC in HK-2 cells. **E**,** G**: The level of protein expression and quantitative analysis of MMP-13 in 24w db/m and 24w db/db mice. **F**,** H**: The protein expression level and quantitative analysis of TIMP-1 in 24w db/m and 24w db/db mice. **I**,** J**,** K**: Protein–protein interaction network (PPI), GO and KEGG enrichment predicted the top 10 target genes of MMP-13. **L**,** M**,** N**: Protein–protein interaction network (PPI), GO and KEGG enrichment predicted the top 10 significant target genes of TIMP-1. *Rluc* *vector* empty vector, *WT* wild-type, *Mut* mutant, *NC* scrambled negative control transfection. *P < 0.05, **P < 0.01, ***P < 0.001

The qRT-PCR results showed that the levels of MMP14, MMP13, MMP9, CTGF and TIMP1 were greatly decreased after overexpression of miR-1470 or miR-4483 and that these target genes were increased after inhibition of miR-1470 or miR-4483 (Fig. 6E–F). Therefore, the wild-type and mutant plasmids were established by a double luciferase reporter vector (Fig. 6G–J). MMP13, MMP9, CTGF, TIMP1 wild-type, mutant plasmids and miR-1470 or miR-4483 mimics, mimic NC were transfected into HK-2 for 48 h to 72 h, and the fluorescence activity was detected by dual-luciferase reporter gene system. The results showed that when co-transfected with rluc-MMP13-WT, rluc-TIMP1 -WT plasmid, the addition of miR-1470 mimic, miR-4483 mimic reduced the luciferase activity compared with the negative control group. In conclusion, it indicates that miR-1470 can bind directly to MMP13, while miR-4483 binds to TIMP1 and thus is involved in renal fibrosis (Fig. 6 K, L). Figure 7 A–D showed that the levels of MMP13 and TIMP1 were decreased after overexpression of miR-1470 or miR-4483, whereas transfection of miR-1470 or miR-4483 inhibitors into HK-2 showed the opposite trend. In addition, the western blot results of the kidney tissue of DN mice showed that the expression of MMP13 was decreased in the kidney tissue of 24w db/db mice compared with that of 24w db/m mice, while the expression level of TIMP1 decreased.

Finally, the protein–protein interaction network (PPI), GO, and KEGG enrichment were used to predict the top 10 significant target genes of MMP13 and TIMP1, which indicate that MMP13 and TIMP1 are closely related to the synthesis of extracellular matrix and collagen and thus participating in the process of renal fibrosis (Fig. 7I–N).

## Discussion

MicroRNAs are conserved, endogenous and short RNA molecules whose typical role is to regulate the expression of their target proteins through mRNA degradation or translation inhibition (Makeyev and Maniatis 2008). MicroRNAs can regulate many cellular processes, including cell type differentiation, migration, survival and proliferation, which play an important role in cell physiology and pathophysiology. Many microRNAs are involved in the occurrence and development of chronic or acute nephropathy in patients or animal models (Mahtal et al. 2022). For instance, miR-29 reduces renal interstitial fibrosis, while miR-382 and miR-21 lead to increased fibrosis (Qin et al. 2011; Chau, et al. 2012; Kriegel et al. 2012). In the diabetic glomerular system, miR-192 mediates the effect of TGF-β1 on vascular cells and glomerular injury (Kato et al. 2007). The miR-30 family protects against podocellular injury and proteinuria (Wu et al. 2015).In addition to these above functions, miRNAs can be regarded as prospective disease markers and contribute to the development of precision medicine. For example, a study of 112 non-small cell lung cancer patients showed characteristics of 5 microRNAs that predict disease progression (Yu et al. 2008). Another study of 109 polyps undergoing colonoscopy found microRNA expression profiles of four different histopathological polyps compared with normal mucosa (Yu et al. 2008).

A previous study on esophageal cancer showed that miR-1470 was located at 19p13.12, revealing that miR-1470 was upregulated in ESCC tissues. Moreover, functional experiments showed that downregulation of miR-1470 significantly inhibited the proliferation of ESCC cells and decreased the level of CCNE1, a cell cycle regulation gene. The results indicated that upregulation of miR-1470 expression could promote cell proliferation and accelerate cell cycle transition (Mei et al. 2017). Meanwhile, Nie et al. confirmed that miR-1470 induces the upregulation of p27 by targeting C-Jun-mediated lapatinib (Nie et al. 2015), but the expression, role, and potential molecular mechanism of miR-1470 in the progression of CKD remains to be determined. Importantly, miR-1470 and miR-4483 identified in this study have not been previously detected in kidney disease and may represent novel disease mechanisms worthy of further investigation. As seen in the study of this work, miR-4483 was downregulated in renal tubules of patients with DN and FSGS, whereas miR-1470 was upregulated, so its role in renal fibrosis and related mechanisms can be explored in an innovative way.

Fibrosis is a basic connective tissue lesion defined as an increase in fibrous extracellular matrix (ECM) in a tissue or organ. Matrix metalloproteinases (MMPs) are the major proteases regulating the translocation of ECM, therefore, they are thought to be significant in tissue remodeling in the fibrosis process. Physiological tissue remodeling is the result of the imbalance between normal synthesis and degradation of ECM, which is largely controlled by MMPs/TIMP imbalance (Robert et al. 2016). MMPs are zinc-binding proteins that have been shown to play a role in tumor cell metastasis due to their ability to degrade ECM. Overexpression of MMPs can aggravate the digestion and degradation of various components of ECM (Wang et al. 2018). MMPs and tissue inhibitors of metalloproteinase (TIMP) are two important factors in regulating the synthesis and degradation of ECM. MMPs mainly degrade the main components of the extracellular matrix, collagen I and III, and TIMP1 can inhibit the activity of MMPs (Nagase and Brew 2003).

In this study, we also identified that the regulation of fibrosis via targeting MMP13 and TIMP1 may be the mechanisms by which miR-1470 and miR-4483 are involved in the process of renal fibrosis. By loss-of- and gain-of-function regulation in vitro, our results confirmed that miR-1470 inhibited fibrosis formation, whereas miR-4483 promoted it. As demonstrated by qRT-PCR, western blot, and luciferase reporter assay, MMP13 and TIMP1 were found to be the true target genes of miR-1470 and miR-4483, respectively. Our study showed that miR-1470 could bind to the 3'-UTR of MMP13, inhibiting its expression and reducing the degradation of the extracellular matrix, which promoted the process of renal fibrosis. Meanwhile, miR-4483 was decreased in fibrotic kidneys and HG or TGF-β1-treated HK-2 cells, whereas TIMP1 was inhibited by miR-4483 at both mRNA and protein levels, which may be the underlying mechanism that miR-4483 is involved in renal fibrosis, which requires further research.

There are some defects in the present study. First, the levels of miR-1470 and miR-4483 were not artificially inhibited or overexpressed in DN and FSGS mice models so as to observe the therapeutic effect of downregulation of miR-1470 or upregulation of miR-4483 in vivo on renal fibrosis. Second, the dual-luciferase reporter gene showed that the addition of miR-1470, miR-4483 mimics could reduce the luciferase activity of MMP13, TIMP1 in HK-2 cells. However, after co-transfection of miR-1470, miR-4483 mimics with the mutant MMP13 or TIMP1 plasmid at the binding site, the fluorescence activity of luciferase did not change significantly. In summary, the binding sites of MMP13 and miR-1470, TIMP1, and miR-4483 should be re-selected to construct mutant plasmids for further dual-luciferase analysis. Third, the heterogeneity of the MCD, FSGS and DN samples makes it complex to clarify the importance of dysregulated miRNAs. Comprehensive studies of a single disease at different stages are likely to provide more intriguing data. Finally, the detailed mechanisms by which miR-1470 and miR-4483 participate in specific types of kidney diseases need to be further explored.

## Conclusion

The present study identifies newly dysregulated miRNA profiles related to fibrosis kidneys. Hsa-miR-1470-3p and hsa-miR-4483-3p are demonstrated to be involved in kidney fibrosis by regulation of MMP13 and TIMP1, respectively. Our results may represent a promising research direction for renal disorders and help identify new biomarkers and therapeutic targets for chronic kidney disease.

## Supplementary Information

Below is the link to the electronic supplementary material.

Supplementary file1 (DOCX 18 KB)

## Data Availability

All data used or analyzed in this study is available from the corresponding author upon reasonable request.
