# Supplementary material for: Identification and Functional Mechanism Verification of Novel MicroRNAs Associated with the Fibrosis Progression in Chronic Kidney Disease
Source: Biochem Genet. 2024 Feb 5;62(6):4472–93. doi: 10.1007/s10528-024-10688-7 (PMC11604686; doi:10.1007/s10528-024-10688-7)
Supplement: Supplementary file 1 — Supplementary file1 (DOCX 18 KB) [file 10528_2024_10688_MOESM1_ESM.docx]

Supplementary table 1 Inclusive criteria and exclusive criteria

|  | Inclusive criteria | Exclusive criteria |
| --- | --- | --- |
| MCD | 1. ≥18 years old;  2. Clinical diagnosis of nephrotic syndrome;  3. Pathological diagnosis was MCD (light glomerular lesions were mild, tubular atrophy and interstitial fibrosis were not observed; Immunopathology was basically negative. Electron microscopy: MCD), and the pathological score was 0；  4. Normal renal function； | 1.Secondary MCD includes drug-related, infection-related, etc.  2. Familial MCD.  3. Used hormones and/or immunosuppressants before renal biopsy. |
| FSGS | 1. ≥18 years old;  2. Pathological diagnosis was FSGS. Renal tubular atrophy and interstitial fibrosis were observed under light microscope, and the pathological score was ≥2; | 1. Secondary and familial FSGS;  2.Patients complicated with acute renal failure and/or pathological lesion of acute renal tubule interstitium;  3.Pathological damage caused by other etiologies;  4. Used hormones and/or immunosuppressants before renal biopsy. |
| DN | 1.≥18 years old;  2. Clinical diagnosis of diabetes;  3. The pathological diagnosis was DN. Tubular atrophy and interstitial fibrosis were observed under light microscope, and the pathological score was ≥2； | 1. Patients complicated with acute renal failure and/or pathological lesion of acute renal tubular interstitium;  2.Pathological damage caused by other etiologies, including hypertensive kidney damage, hepatitis b-related kidney damage, etc.;  3.Used hormones and/or immunosuppressants before renal biopsy； |

Supplementary table 2 miRNA array analysis of CKD patients

| Group | Gender | Age | Hospital number | Pathological number | Nephropuncture time |
| --- | --- | --- | --- | --- | --- |
| MCD | Male | 27 | 828564 | 12852 | 2009-12-8 |
|  | Female | 28 | 848024 | 13408 | 2010-6-8 |
|  | Female | 23 | 867185 | 13928 | 2010-12-16 |
|  | Male | 37 | 873379 | 14078 | 2011-2-22 |
| FSGS | Male | 43 | 831642 | 12966 | 2010-1-12 |
|  | Male | 26 | 851452 | 13505 | 2010-7-13 |
|  | Female | 46 | 861785 | 13778 | 2010-10-14 |
|  | Male | 23 | 872661 | 14041 | 2011-1-28 |
| DN | Female | 40 | 704530 | 9089 | 2006-5-30 |
|  | Female | 37 | 826577 | 12964 | 2010-1-12 |
|  | Male | 49 | 878361 | 14174 | 2011-3-31 |
|  | Male | 56 | 883511 | 14286 | 2011-5-12 |

Supplementary table 3：The sequence of microRNA mimic, inhibitor, mimic NC, inhibitor NC

| hsa-miR-4483-3p mimic | 5'-GGGGUGGUCUGUUGUUG-3' | 5'-CAACAACAGACCACCCC-3' |
| --- | --- | --- |
| hsa-miR-4483-3p inhibitor | 5'-CAACAACAGACCACCCC-3' |  |
| hsa-miR-1470-3p mimic | 5'-GCCCUCCGCCCGUGCACCCCG-3' | 5'-CGGGGUGCACGGGCGGAGGGC-3' |
| hsa-miR-1470-3p inhibitor | 5'-CGGGGUGCACGGGCGGAGGGC-3' |  |
| mimic NC | 5'-UUUGUACUACACAAAAGUACUG-3' | 5'-CAGUACUUUUGUGUAGUACAAA-3' |
| inhibitor NC | 5'-CAGUACUUUUGUGUAGUACAAA-3' |  |

Supplementary table 4：qRT-PCR primers sequences are used in this study.

| primer | Sequence(5’-3’) |
| --- | --- |
| mus-COL3A1-3-F | CTGTAACATGGAAACTGGGGAAA |
| mus-COL3A1-3-R | CCATAGCTGAACTGAAAACCACC |
| mus-COL1A1-3-F | GATGACGTGCAATGCAATGAA |
| mus-COL1A1-3-R | CCCTCGACTCCTACATCTTCTGA |
| mus-a-SMA-F | GTCCCAGACATCAGGGAGTAA |
| mus-a-SMA-R | TCGGATACTTCAGCGTCAGGA |
| mus-FN-3-F | TACCAAGGTCAATCCACACCCC |
| mus-FN-3-R | CAGATGGCAAAAGAAAGCAG |
| mus-β-actin-F | AGAGGGAAATCGTGCGTGAC |
| mus-β-actin-R | CAATAGTGATGACCTGGCCGT |
| Hsa-COL3A1-3-F | GGAGCTGGCTACTTCTCGC |
| Hsa-COL3A1-3-R | GGGAACATCCTCCTTCAACAG |
| Hsa-COL1A1-3-F | ATCAACCGGAGGAATTTCCGT |
| Hsa-COL1A1-3-R | CACCAGGACGACCAGGTTTTC |
| Hsa-alpha-SMA-3-F | CTGTTCCAGCCATCCTTCAT |
| Hsa-alpha-SMA-3-R | TCATGATGCTGTTGTAGGTGGT |
| Hsa-FN-3-F | GAGAATAAGCTGTACCATCGCAA |
| Hsa-FN-3-R | CGACCACATAGGAAGTCCCAG |
| Hsa-β-actin-F | CCCTGAAGTACCCCATCGAGCACG |
| Hsa-β-actin-R | GGTCATCTTCTCGCGGTTGGCCT |
| Hsa-MMP-14-F | CTCAGACCTCGCTGGTAAAG |
| Hsa-MMP-14-R | GGTCAGAGTTCAGAGGTTAAGG |
| Hsa-MMP-2-F | GGCACCCATTTACACCTACA |
| Hsa-MMP-2-R | CCAAGGTCAATGTCAGGAGAG |
| Hsa-MMP-13-F | AGCATCTGGAGTAACCGTATTG |
| Hsa-MMP-13-R | CCCGCACTTCTGGAAGTATT |
| Hsa-TIMP1-F | TCCCAGATAGCCTGAATCCT |
| Hsa-TIMP1-R | CTGCTGGGTGGTAACTCTTTAT |
| Hsa-MMP-9-F | GGGCTTAGATCATTCCTCAGTG |
| Hsa-MMP-9-R | GCCATTCACGTCGTCCTTAT |
| Hsa-CTGF-F | AGGACCAAACCGTGGTTGG |
| Hsa-CTGF-R | GGCTCTAATCATAGTTGGGTCTGG |
